# Supplementary material for: A Facile and Highly Efficient Approach to Obtain a Fluorescent Chromogenic Porous Organic Polymer for Lymphatic Targeting Imaging
Source: Molecules. 2022 Feb 25;27(5):1558. doi: 10.3390/molecules27051558 (PMC8911811; doi:10.3390/molecules27051558)
Supplement: Supplementary file 1 [file molecules-27-01558-s001.zip › molecules-1581941-supplementary.pdf]

Supporting information

A Facile and Highly Efficient Approach to Obtain a Fluorescent Chromogenic Porous Organic Polymer for Lymphatic Targeting Imaging

Man Duan,[1] Dongmei Han,[1] Nan Gao,[2,*] Wenbin Shen,[3] Kun Chang,[3] Xinyu Wang,[1] and Jianshi Du [1, *]

[1] China-Japan Union Hospital of Jilin University, Key Laboratory of Lymphatic Surgery Jilin Province, Jilin Engineering Laboratory for Lymphatic Surgery Jilin Province, Changchun 130031, P. R. China; duanman19@mails.jlu.edu.cn (M. D.); handm@jlu.edu.cn (D. H.); wangxinyu18@mails.jlu.edu.cn (X. W.); dujs@jlu.edu.cn (J. D.)

[2] Key Laboratory of Polyoxometalate and Reticular Material Chemistry of Ministry of Education and Faculty of Chemistry, Northeast Normal University, Changchun 130024, P.R. China; gaon320@nenu.edu.cn (N. G.)

[3] Department of Lymphology, Beijing Shijitan Hospital, Capital Medical University, Beijing 100038, P. R. China; shenwb@bjsjth.cn (W. S.); chang0213@sina.com (K. C.)

* Correspondence: gaon320@nenu.edu.cn (N. G.); dujs@jlu.edu.cn (J. D.)

Materials

1,3,5-Tris(4-bromophenyl)benzene and 2,5-diethynylaniline were received from TCI. Copper iodide and tetrakis(triphenylphophine)palladium were got from Sigma-Aldrich. Other chemicals and solvents were purchased from commercial suppliers and used without further purification. All reactions were performed under a purified nitrogen atmosphere.

Method

FT-IR measurements were performed on the Nicolet IS50 Fourier transforms infrared spectrometer. TGA was measured on the METTLER-TOLEDO TGA/DSC 3 analyzer at the 10 $^{o}C \cdot min^{-1}$ heating rate in air atmosphere. N_2-adsorption isotherms and pore size distribution were obtained at 77 K using an Autosorb iQ2 adsorptometer, Quantachrome Instrument. SEM imagery was implemented on the field emission scanning electron microscopy (FE-SEM, SU-8010, Hitachi). PXRD measurements were carried out on the Rigaku SmartLab X-ray diffractometer with Cu-Kα radiation (40 kV, 30 mA, λ = 1.5418 Å). ^{13}C CP/MAS solid-state NMR spectra were obtained using a Bruker Avance III model 400 MHz NMR spectrometer at a MAS rate of 5 kHz. UV-Vis spectra were recorded on a Cary 500 UV-Vis-NIR spectrophotometer.

Process

Take 5 mg of commercially available hyaluronic acid (abbreviated as HA, derived from Streptococcus equi) (Compared to the other half of the amino groups remaining on the surface of NH_2-POP, the amount of hyaluronic acid is in large excess, so that all amino groups on the surface of NH_2-POP can be completely reacted in the next step), 0.103 g DCC (about 0.5 mmol, large excess), 0.0575 g NHS (about 0.5 mmol, large excess) was dissolved in 5 ml water and stirred at room temperature for 24 hours to obtain carboxyl activated HA.

The ICG-POP powder was dispersed in 5 ml methanol, then the reaction product in above step was directly added, stirred at room temperature for 8 h, then centrifuged at 10000 rpm/min to collect the precipitation, washed with respective DMF and water for three times to obtain the final ICG-POP-HA nano-tracers.

Experimental details

Cell Culture: MLEC cells, obtained from Procell Life Science&Technology Co.,Ltd. (Wuhan, China), were grown on plates in MEM media containing 10% fetal bovine serum, 1% serum L-glutamate and 1% streptomycin in a humidified atmosphere of 5% CO_2 and 95% air. The media were changed every three days, and the cells were passaged by trypsinization before confluence.

Cellular accumulation and quantification of the fluorescence intensity: MLEC cells, RAW 264.7 cells and CT26 cells were seeded on six-well plates at 1×10^5 cells per

well. After 12 h incubation, cells were treated with ICG or ICG-POP-HA. Then, cells were washed different times with PBS and incubated in fresh medium. The fluorescences of cells were estimated by CLSM (FV3000, Olympus Corporation).

Cytotoxicity Assays: Cell viability was measured by 3-(4,5-dimethylthiazol-2-yl)-2,5-diphenyltetrazoliumbromide (MTT) assays. MLEC cells were seeded at a density of 5000 cells/well on 96-well plates for 24 h. Cells were incubated for 24 h and were treated with the samples with different concentration and then MTT assays were carried out. To determine toxicity, 10 μL of MTT solution was added to each well and the cells were incubated for 4 h. After adding 100 μL DMSO, the absorbance of formazan was read at 490 nm on a SpectraMax M5 microplate reader. Three replicates were done for each treatment group.

Mouse tissue section: All animal studies were conducted in accordance with the principles and procedures outlined in the Guide for the Care and Use of Laboratory Animals and were approved by the Institutional Animal Care and Use Committee of Jilin University. After sacrificed, mouse leg tissues were harvested and were sectioned into 10 μm slices. Slices were incubated with rat anti-mouse LYVE-1 antibody (modified with Rhodamine B) and ICG-POP-HA (1 mg/ml) at room temperature for 2 h. Between each step, the slices were gently washed 5 times with 20 mM PBS solution for 5 min each time. The staining observation was done with the confocal laser scanning microscopy (FV3000, Olympus Corporation).

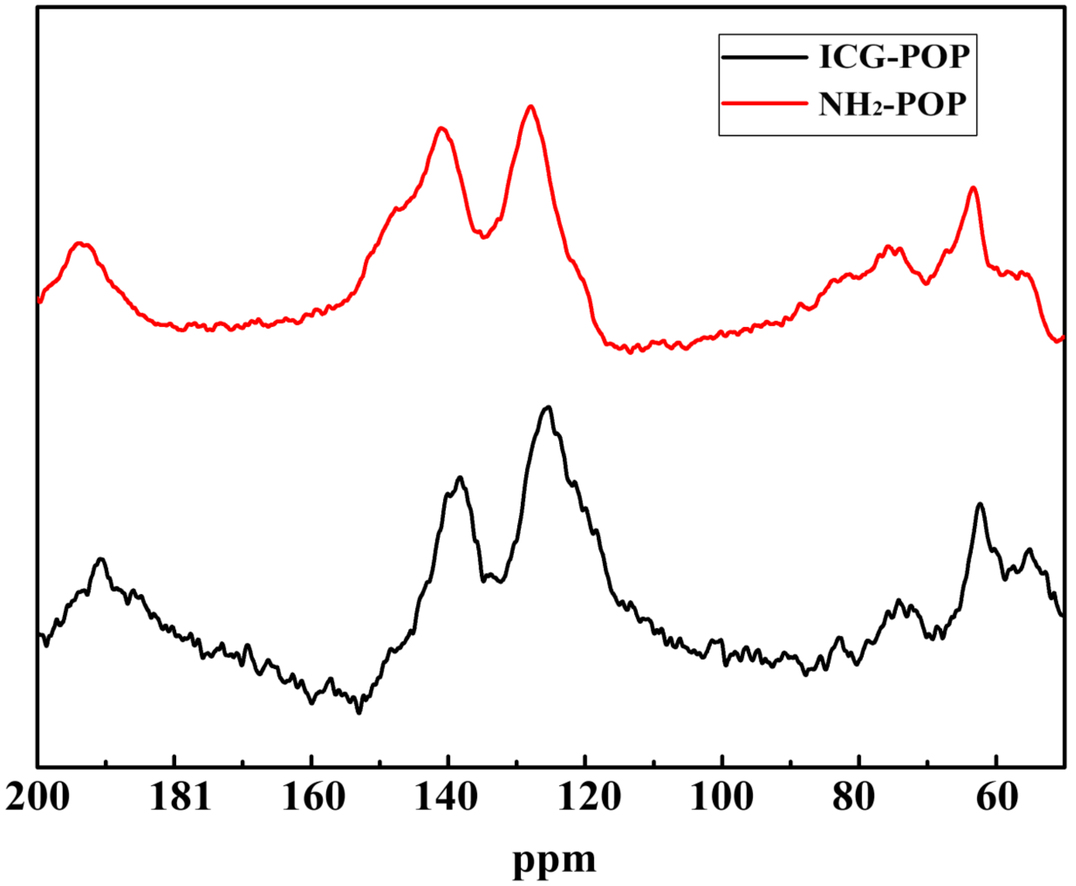

Figure S1. Solid-state ^{13}C NMR spectra of POP solids.

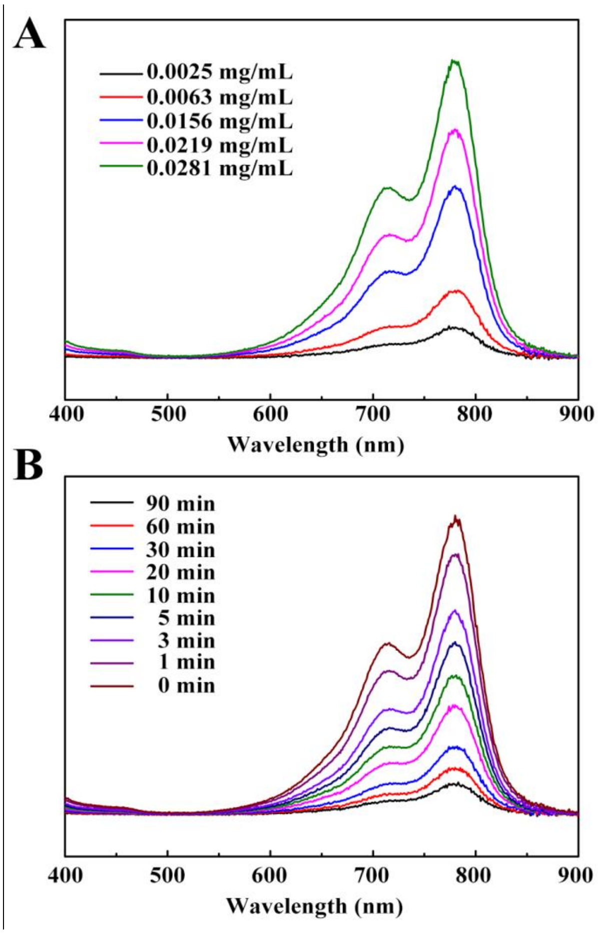

Figure S2. (a) The UV spectra records different concentrations of ICG molecule as the standard. (b) The UV spectra records the concentrations of ICG molecule through time after treated by 10 mg POP solid.

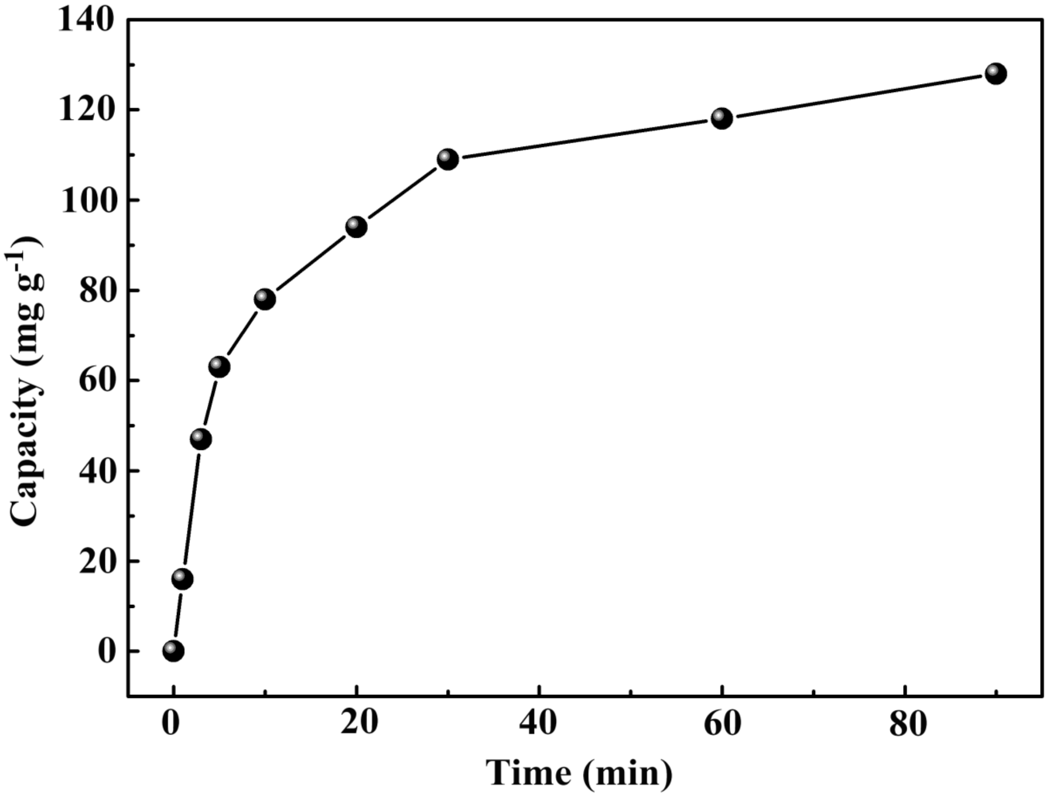

Figure S3. The ICG uptake of NH_2-POP according to *Beer–Lambert's law*. The highest ICG uptake of NH_2-POP is calculated to be ~128 mg g^{-1} in 90 minutes. Accordingly, the ICG uptake on the ICG-POP-HA is calculated to be ~122 mg g^{-1}.

Beer–Lambert's law: When the concentration is controlled within certain bounds, it presents a linear relationship with optical intensity, which is calculated by equation (1):

$$A = \log\frac{1}{T} = Kbc$$

where *A* is the absorbance, *T* represents the transmittance, *K* is the molar absorption coefficient, *b* is the thickness of absorbing layer, and *c* is the concentration of substrate.

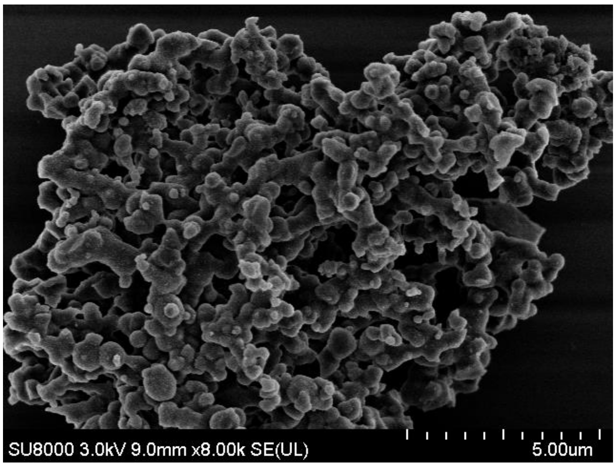

Figure S4. SEM image for ICG-POP-HA.

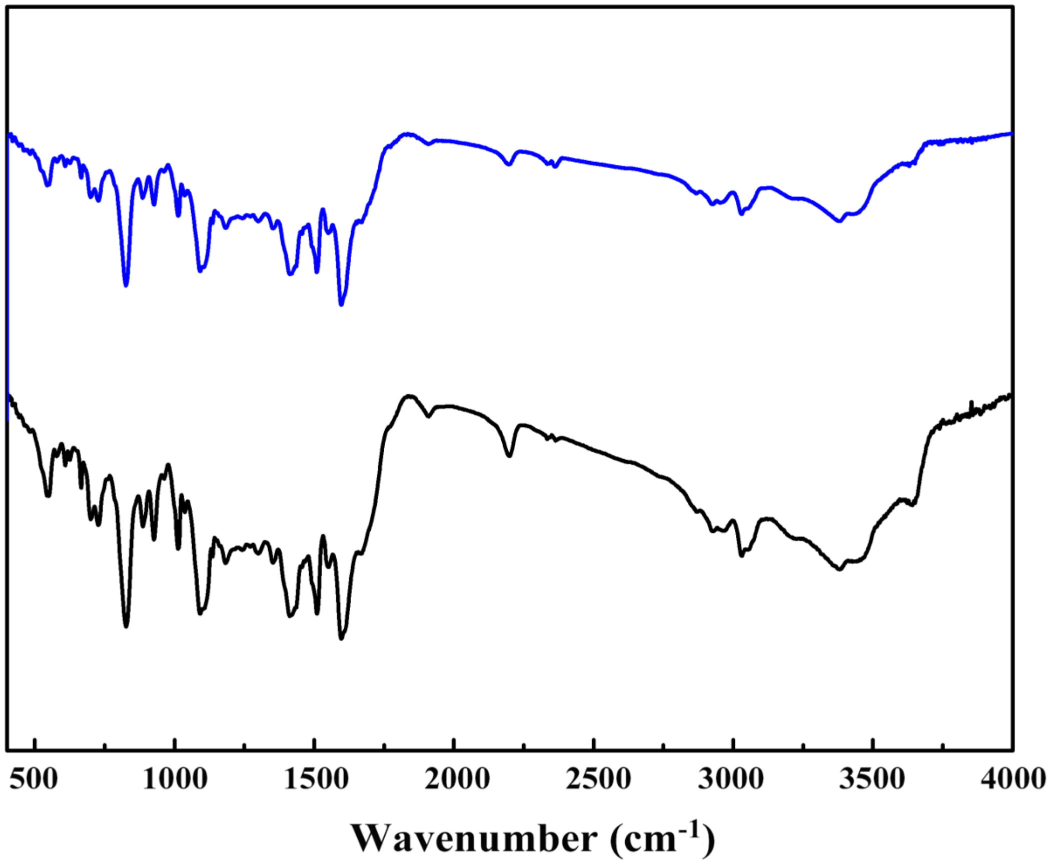

Figure S5. FTIR spectra of ICG-POP before (black) and after (blue) heated at 200 degrees for 24 hours. The infrared spectrum of the ICG-POP sample did not change significantly.

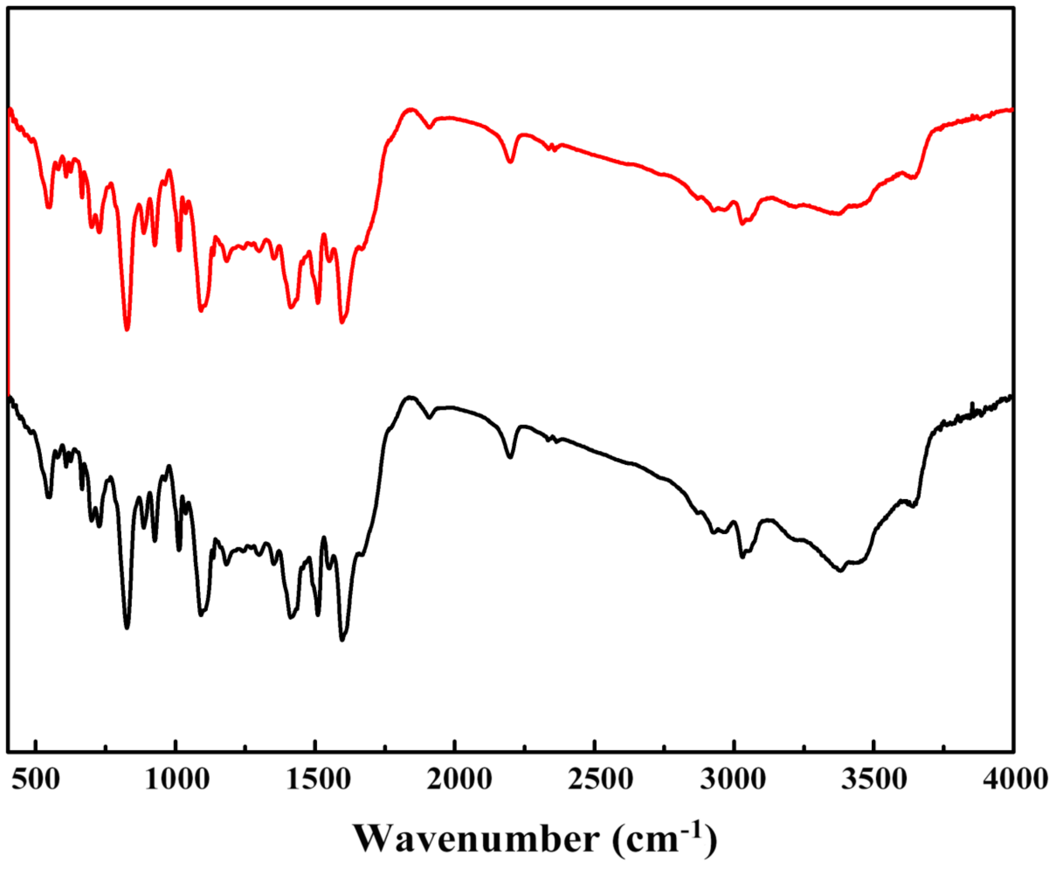

Figure S6. FTIR spectra of ICG-POP before (black) and after (red) soaked into different organic solvents. Suffering from various solvents, including dichloromethane, acetone, ethanol, etc., the infrared spectrum of the ICG-POP sample did not change significantly.

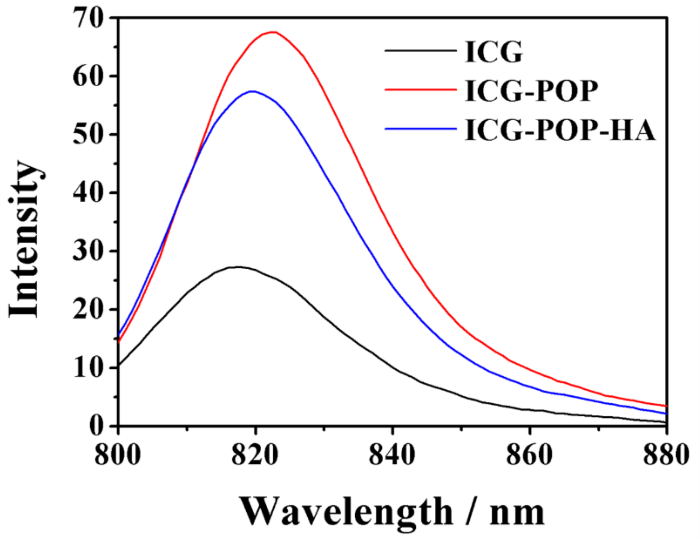

Figure S7. Fluorescence emission spectra of NH_2-POP (black), ICG-POP (red) and ICG-POP-HA (blue) dispersed in water (0.25 mg mL^{-1}).

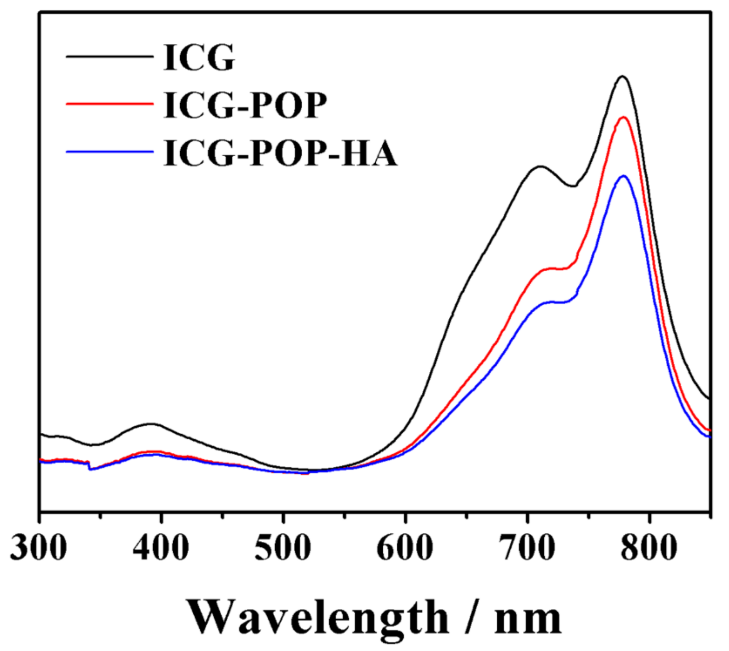

Figure S8. UV-Vis spectra of ICG (black), ICG-POP (red) and ICG-POP-HA (blue).

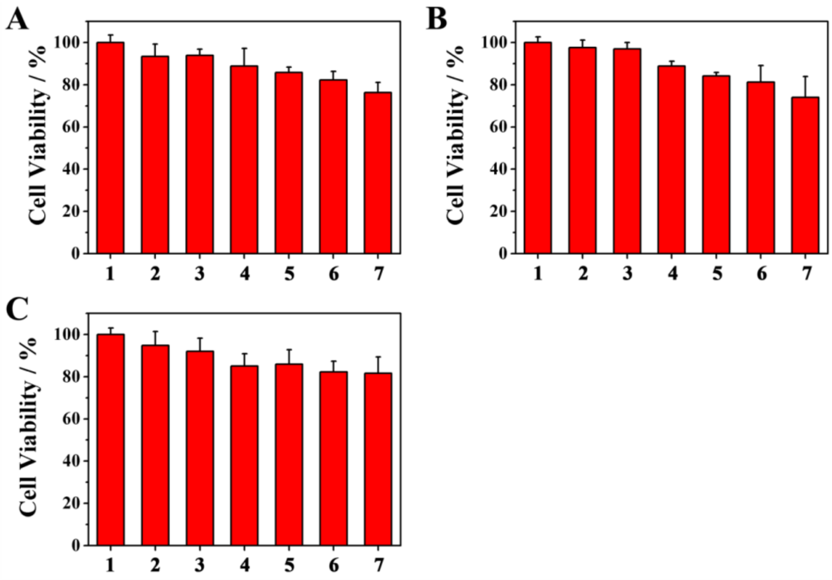

Figure S9. The cell toxicity of different concentrations of ICG@POP-HA co-incubated with RAW cells (A), CT26 cells (B) and MLEC (C) for 24 h. Each experiment had been repeated three times. Error bars indicate ±s.d. 1: control (RAW cells only); 2: add 0.1 mg/ml ICG@POP-HA; 3: add 0.3 mg/ml ICG@ POP-HA; 4: add 0.5 mg/ml ICG@POP-HA; 5: add 1.0 mg/ml ICG@ POP-HA; 6: add 3.0 mg/ml ICG@ POP-HA; 7: add 5.0 mg/ml ICG@ POP-HA.

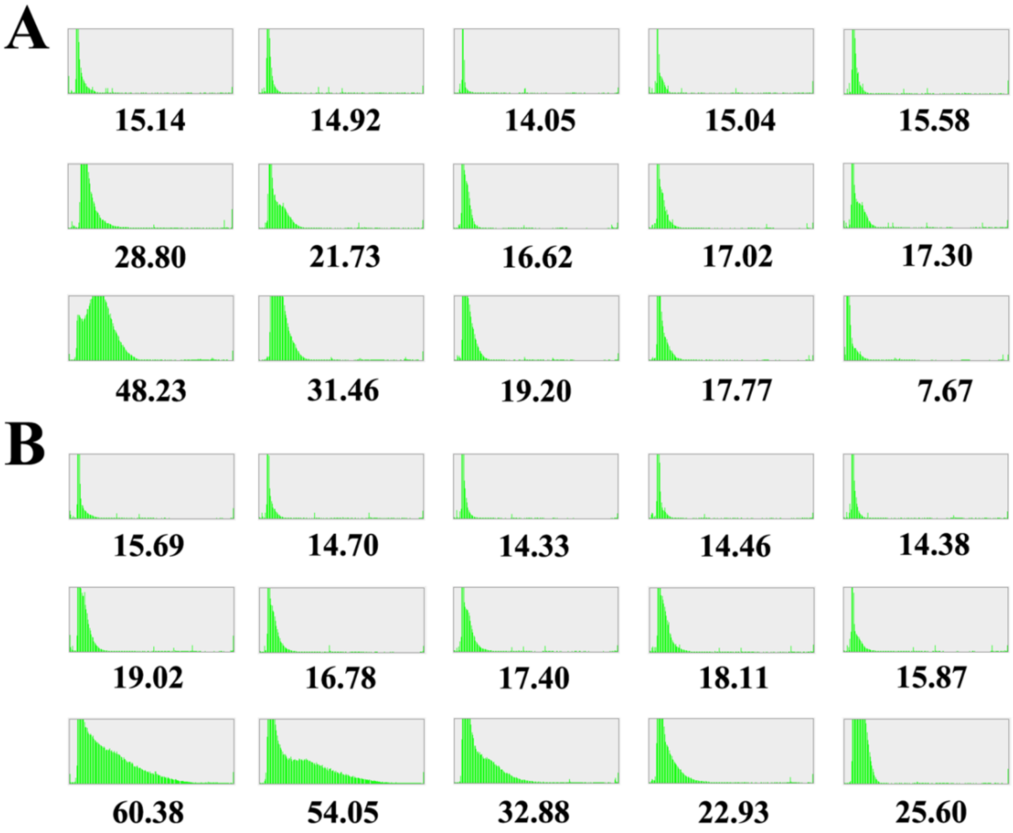

Figure S10. The average strength of the green fluorescence channel in Figure 5 evaluated using Adobe Photoshop CS3 software.

Table S1. CHN element analysis.

		C	H	N	S
NH_2-POP	Experimental	90.73%	4.11%	3.75%	
	Calculated	92.86%	4.37%	2.77%	--
ICG-POP	Experimental	91.04%	3.27%	3.08%	2.55
	Calculated	92.19%	3.50%	2.61%	1.70%
